# Seabirds’ mortalities in Antarctica caused by HPAIV: skuas as a sentinel species

**DOI:** 10.1017/S0950268826101733

**Published:** 2026-06-09

**Authors:** Fabiola León, Claudia Ulloa-Contreras, Eduardo J. Pizarro, Pablo N. Castillo-Torres, Karla B. Díaz-Morales, Elisa Gutiérrez-Iturrieta, Ana Claudia Franco, Francine C. B. Timm, Miguel L. Correa, Magdalena Johow, Christian Mathieu, Carolina Aguayo, Harm van Bakel, Zain Khalil, Lucas Kruger, Elie Poulin, Catalina Pardo-Roa, Juliana A. Vianna

**Affiliations:** 1 Millennium Institute Center for Genome Regulation (CGR), Santiago, Chile; 2 Millennium Institute Biodiversity of Antarctic and Subantarctic Ecosystems (BASE), Santiago, Chile; 3 Center for Oceanology and Antarctic Studies, Venezuelan Institute for Scientific Research (IVIC), Caracas, Venezuela; 4 Pontificia Universidad Católica de Chile, Santiago, Chile; 5 Department of Child and Adolescent Health, School of Nursing, Pontifical Catholic University of Chile, Santiago, Chile; 6 Department of Microbiology, Immunology and Parasitology, Institute for Basic Health Sciences, Universidade Federal do Rio Grande do Sul, Brazil; 7 Servicio Agrícola y Ganadero (SAG), Lo Aguirre, Chile; 8 Center for Research on Influenza Pathogenesis and Transmission (CRIPT), Center of Excellence of Influenza Research and Response (CEIRR) and Department of Genetics and Genomic Sciences, Icahn School of Medicine at Mount Sinai, New York, NY, USA; 9 Departamento Científico, Instituto Antartico Chileno, Punta Arenas, Chile; 10 Facultad de Ciencias, Universidad de Chile, Santiago, Chile; 11 Emory Center of Excellence of Influenza Research and Response, Atlanta, USA; 12 One Health Institute, Faculty of Life Sciences, Universidad Andres Bello, Santiago, Chile; 13 Pontificia Universidad Católica de Chile, Department of Pediatric Infectious Diseases and Immunology, School of Medicine, Santiago, Chile

**Keywords:** Antarctica, skuas mortality, influenza, seabirds, sentinel, HPAIV

## Abstract

Seabirds are at high risk of extinction, and the emergence of highly pathogenic avian influenza virus (HPAIV) represents an additional threat. In 2024, HPAIV A/H5 clade 2.3.4.4b was detected for the first time in the Antarctic Continent in a dead Brown Skua (*Stercorarius antarcticus*), and further evidence suggests that not all species have been equally affected. We conducted a survey of HPAIV-related mortalities across 16 seabird nesting sites along the western Antarctic Peninsula (WAP) during the 2024–2025 breeding season. We report a mortality of 35 adult skuas along the WAP and beyond the Antarctic Polar Circle. Eleven skuas from six different geographic areas were sampled and all were positive to HPAIV A/H5 clade 2.3.4.4b. Phylogenetic analyses revealed that these sequences are closely related to the initial HPAIV A/H5 clade 2.3.4.4b reported in Antarctica, and post-mortem findings suggest that the viral infection was likely the cause of death. No other suspected HPAIV-related mortalities were observed in sympatric species. Our findings represent the southernmost record of skua mortality in Antarctica related to HPAIV A/H5 infection to date, demonstrating the dissemination of 2.3.4.4b clade across several localities. The virus is differentially affecting sympatric seabird species and the skuas may act as sentinel species for influenza surveillance.

## Introduction

Seabirds are among the most threatened vertebrate groups globally [1–3]. While climate change and overfishing have long been recognized as major drivers of their population declines, emerging infectious diseases have recently gained prominence as additional and potentially severe threats. Among these, highly pathogenic avian influenza virus (HPAIV) A/H5 clade 2.3.4.4b has emerged as a major conservation concern due to its capacity to cause mass mortality events in wild bird populations [4–6].

This clade was first reported in Eurasia in 2016 causing sporadic mortality events in poultry and wild birds. In 2020, a genomic reassortment enabled an expansion of both its geographic and host range, triggering large-scale mortality events across Europe, Africa, and North America [7]. Between 2021 and 2022, the virus spread southward through the Pacific and Mississippi migratory flyways, eventually reaching South America and causing unprecedented mass mortality events in seabirds and marine mammals [8–11]. By 2023, mass mortalities had also been documented in the Subantarctic region, affecting multiple seabird species including Gentoo Penguins (*Pygoscelis papua*), Black-browed Albatross (*Thalasarche melanophris*), and Chilean Skuas (*Stercorarius chilensis*) among others [12].

At the western Antarctic Peninsula (WAP), the first suspected cases of HPAIV A/H5 were reported in Adélie Penguins (*Pygoscelis adeliae*) and Antarctic Shags (*Leucocarbo bransfieldensis*) [13], followed by confirmed infection of HPAIV A/H5 clade 2.3.4.4b in a Brown Skua (*Stercorarius antarcticus*) in early 2024 [14]. Since then, additional seabird carcasses, primarily in the northern WAP, have raised concerns that HPAIV outbreaks may exacerbate ongoing declines in seabird populations breeding in this region [15]. These areas are of high conservation importance, supporting large fractions of global seabird populations and several endemic or threatened species [16].

Seabirds appear particularly susceptible to HPAIV, with mortality rates in wild populations reaching up to 90% during outbreaks [6]. The demographic consequences can be especially severe during breeding seasons affected by adult mortality [17, 18] and the high density of colonies further facilitates viral transmission [19]. Nevertheless, species vary in their susceptibility. Evidence from the Northern Hemisphere suggests that skuas may be disproportionately affected [7]. During the 2021–2022 outbreak of HPAIV A5/H5N1 clade 2.3.4.4b, Great Skua (*Stercorarius skua*) experienced severe mortality (69 to 76% population decline) along with drastic reduction in reproductive success [20]. At the Antarctic Peninsula, current evidence suggests that species of the same genus such as Brown and South Polar Skuas (*Stercorarius maccormicki*) appear to be similarly impacted [21]. Their scavenging behaviour and extensive migratory ranges may also facilitate viral spread across regions, underscoring the need for active HPAIV surveillance in these species [21, 22].

Recent genomic analyses indicate that many skuas in the Antarctic Peninsula are admixed individuals resulting from hybridization between South Polar and Brown Skuas, forming two dominant genomic clusters: one with higher South Polar ancestry (70–96%) towards the southern Peninsula and another with higher Brown Skua ancestry (56–75%) towards the northern Peninsula, South Georgia, and Bouvet Island [23]. Because hybrids often exhibit intermediate morphologies, reliance on phenotypic traits alone may lead to misidentifications [24], increasing the risk of inaccurate species classifications in field surveys.

Given the substantial demographic impact of HPAIV A5/H5N1 clade 2.3.4.4b elsewhere [9, 11] and the similar vulnerability observed in *Stercorarius* species across both hemispheres [20, 25], there is an urgent need to document and understand outbreak dynamics in Antarctic populations.

Here, we present a systematic survey of HPAIV-associated mortality in seabirds along the WAP during the 2024–2025 breeding season, with a particular focus on skuas. Our surveillance, conducted between November 2024 and January 2025, included behavioural assessments to identify signs consistent with HPAIV infection and field necropsies of all recovered carcasses, from which samples were collected for viral detection. By integrating field observations, mortality records, H5 antibodies detection, and phylogenetic viral analyses, our study provides urgently needed baseline data for evaluating the unfolding outbreak in Antarctica. These results are critical not only for understanding the immediate conservation impacts on key seabird species, but also for informing early-warning systems, biosecurity protocols, and long-term monitoring strategies under a rapidly changing disease landscape.

## Materials and methods

### Ecological surveillance strategy, site selection, and sampling effort

Three field expeditions were conducted during the 2024–2025 austral summer season. First, a long-term survey has been performed at Harmony Point, Nelson Island (62.305650 S°, 59195120 W°), between 23 November 2024 and 11 January 2025. Additionally, two vessel-based surveys with landings at multiple sites were performed, one aboard the Betanzos vessel from 3 January 2025 to 16 January 2025, and another aboard the Karpuj vessel from 14 January 2025 to 15 January 2025. For this timing, most of the seabirds at the Antarctic Peninsula are expected to be at incubation – hatching or early chick rearing.

For the stationary survey, annual research activities have been performed in this area, which allowed us to include the HPAIV surveillance activities. For the vessel-based surveys, landings were focused on areas along the WAP where a medium to high risk of HPAIV introduction had been previously assessed [21, 22] as well as those that were accessible for the vessel. Priority was given to skua populations near areas hosting high-density breeding colonies including *Pygoscelis* penguins and Antarctic Shags. Skuas near nesting colonies of Southern Giant Petrels (*Macronectes giganteus*) were also included. At each location, efforts were made to maximize area coverage, considering available sampling time, accessibility, and weather conditions.

**Figure 1.**
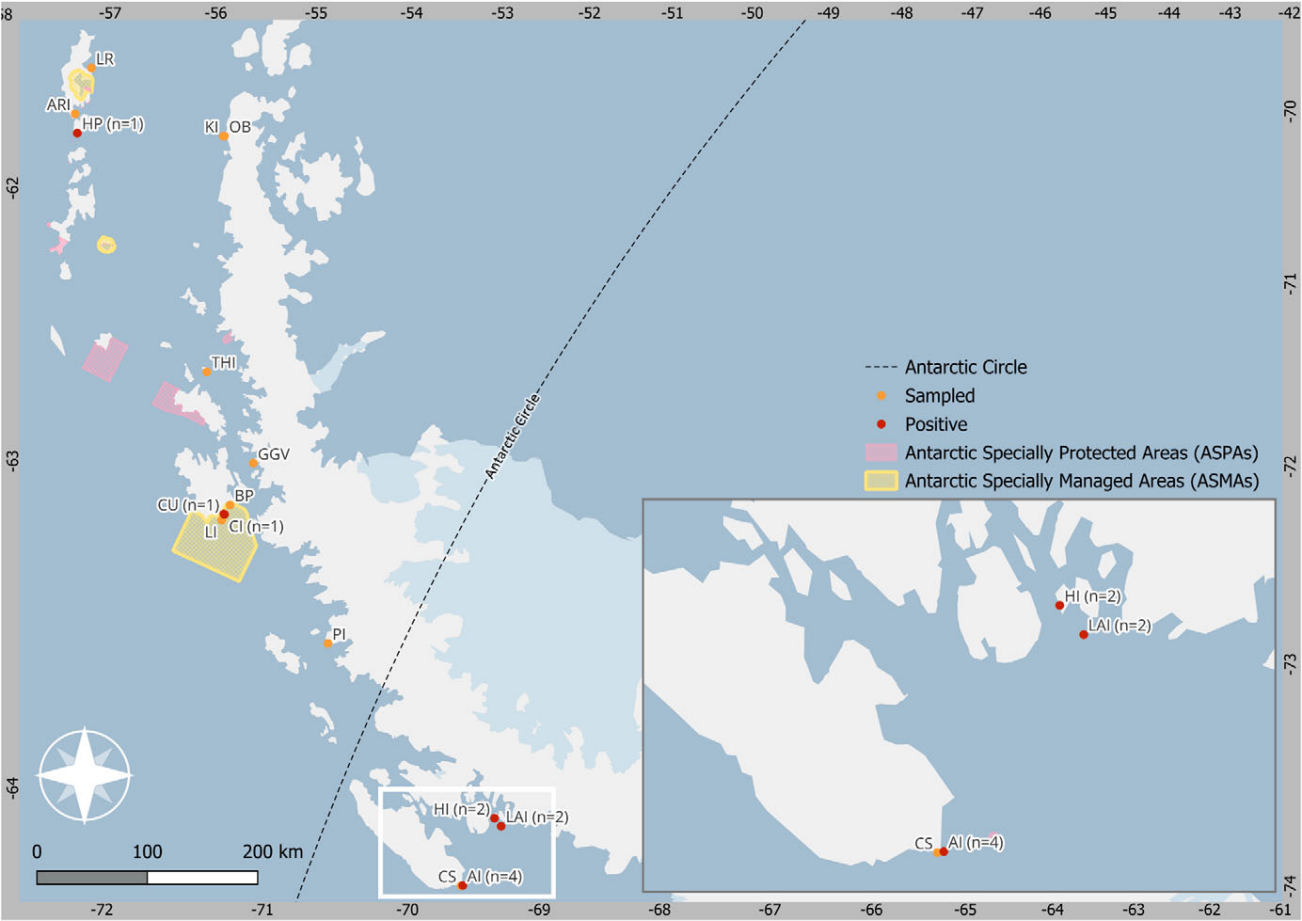
Sampling sites across the Antarctic Peninsula. Sampling sites from north to south: Lions Rump (LR), Ardley Island (ARI), Harmony Point (HP), Kopaitic Island (KI), Bernardo O’Higgins Base (OB), Two Hummock (THI), Cuverville Island (CU), Lichtfield Island (LI), Cormorant Island (CI), Biscoe Point (BP), Gabriel Gonzalez Videla Base (GGV), Perch Island (PI), Carvajal Station (CS), Avian Island (AI), Horseshoe Island (HI), Lagotellerie Island (LAI). Mortalities and positive HPAIV cases are denoted with red stars, while no mortalities or suspected cases are indicated with yellow circles. Six of the localities visited, Ardley Island (ARI), Harmony Point (HP), Litchfield Island (LI), Biscoe Point (BP), Avian Island (AI) and Lagotellerie (LAI) have ASPA or ASMA categories and all of them are Important Bird Areas (IBA). Full list of abbreviations in Supplementary Table 1. Figure 1. long description.

In total, 16 geographic areas were surveyed along the WAP between 62.132683 S°–67882431 S° latitude and 58122040 W°–67399990 W° longitude. The surveyed sites included several Antarctic Specially Protected Areas (ASPA) such as Biscoe Point (ASPA No. 139), Harmony Point (ASPA No. 133), Litchfield Island (IBA criteria A4i; ASPA No. 115), and Avian Island (IBA criteria A1, A4i, A4ii, A4iii; ASPA No. 117). Additional locations between King George Island and the WAP (Table 1 and Figure 1).

**Table 1.** Sampling sites with positive cases of HPAIV H5N1 clade 2.3.4.4b in dead adult skuas Table 1. long description.

Site name	Decimal latitude	Decimal longitude	Sp / Skua ID	Obtained swabs	Ct value (M segment)	Ct value Pathogenic H5 clade 2.3.4.4b	Subtype by nanopore
Harmony Point (HP)	62.30565°S	59.19512°W	BS-1	Orotracheal	24.368	26.384	NA
				Cloacal	27.738	29.587	NA
Cuverville Island (CU)	64.683247°S	62.626093°W	SP-1	Brain	18.942	19.516	H5N1
				Orotracheal	25.224	24.634^a^	*
				Cloacal	26.841	29.851	*
Cormorant Island (CI)	64.7999995°S	63.9666669°W	SP-2	Orotracheal	24.748	22.165	*
				Cloacal	24.507	27.062^a^	H5N1^b^
Avian Island (AI)	67.773099°S	68.894866°W	SP-3	Brain	21.532	20.596	NA
				Orotracheal	22.693	23.360^a^	H5N1
				Cloacal	18.394	19.230	H5N1
	67.773099°S	68.894866°W	SP-4	Brain	20.045	20.199^a^	*
				Orotracheal	22.821	25.327	NA
	67.773099°S	68.894866°W	SP-5	Brain	31.279	27.482	NA
				Orotracheal	26.864	30.121^a^	*
				Cloacal	32.401	30.867	NA
	67.769744°S	68.890579°W	SP-6	Brain	13.976	12.962^a^	H5N1
				Orotracheal	22.375	22.974	H5N1
Horseshoe Island (HI)	67.807993°S	67.295349°W	SP-7	Orotracheal	17.904	19.516	NA
				Cloacal	23.339	22.327^a^	H5N1
	67.807993°S	67.295349°W	SP-8	Orotracheal	26.148	22.175^a^	*
				Cloacal	29.325	29.883	NA
Lagotellerie Island (LAI)	67.882431°S	67.399990°W	SP-9	Orotracheal	36.503	36.480	NA
				Cloacal	27.632	27.144^a^	*
	67.882431°S	67.399990°W	SP-10	Orotracheal	22.624	22.565^a^	*

BS, Brown Skua; NA, sample was not sequenced; SP, South Polar Skua. Notice that not all carcasses had sufficient tissue for the collection of all swab types.

a Sample was confirmed by SAG.

b Incomplete.

* No genomic amplification at multi-segment PCR, not positive to amplify the viral genome, or only M and NS segments are available.

Since all individuals from the Antarctic Peninsula are introgressed among the three species of skuas, here we refer to the species according to the higher percentage of hybridization, South Polar and Brown Skuas. Therefore, the individuals were classified according to the higher genomic proportion reported in the same localities or nearby areas included in this study [23]. At each site, visual inspection of seabirds and colonies was conducted to assess the nesting activity, detect mass mortality events, or dead individuals with evidence of rapid death progression. Also, clinical signs consistent with HPAIV infection were assessed in alive seabirds such as lack of coordination, lethargy, loss of balance, opisthotonus, and respiratory distress [20].

**Figure 2.**
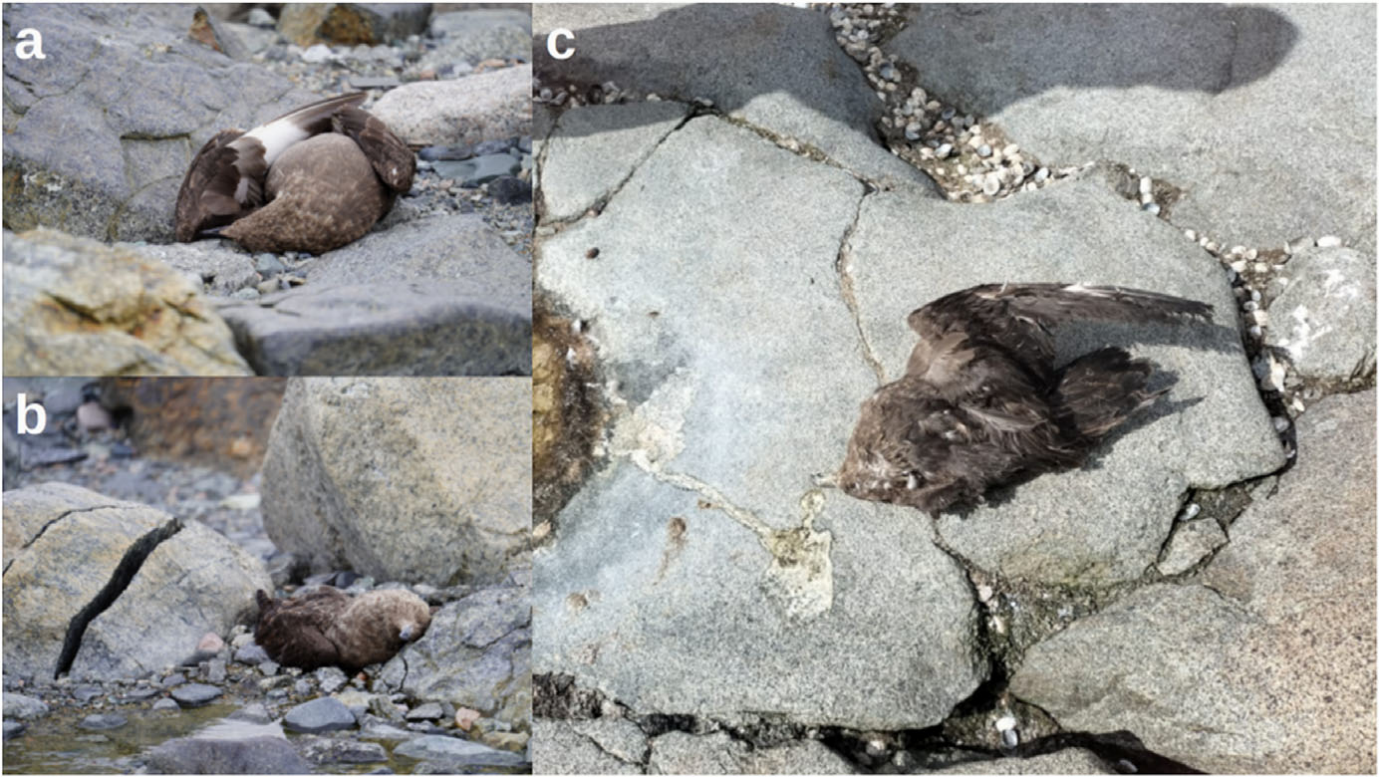
Several dead skuas were observed in pairs near the reported nesting sites in the breeding and rearing areas of the IBA. (a–b) Both sampled birds in Horseshoe Island showed neck position indicating a possible opisthotonus sign at the time of death. Birds had no sign of predation and had well-preserved plumage. (c) Some animals exhibited dry vomit around the bill. Figure 2. long description.

For dead birds, orotracheal, cloacal, brain, or lung swabs were collected for HPAIV detection. Swabs were stored in microtubes, preserved in the Copan eNAT® System, DNA/RNA Shield™, or InhibiSURE™, and then stored in 4 °C, −20 °C, or in liquid nitrogen until analysis. Carcasses were observed to detect abnormal postures suggestive of a death caused by HPAIV infection, such as torticollis posture (twisted) (Figure 2a,b). Necropsies were conducted only on fresh, intact avian carcasses that showed no signs of scavenging or advanced decomposition, to identify macroscopic lesions consistent with HPAIV infection. Body condition was assessed for all carcasses using a five-point fat scoring system to estimate the subcutaneous fat [26]. External palpation and visual inspection were used to detect traumatic injuries and to infer if the bird had recently died. Severely ill agonizing birds were not handled until their death.

### Biosecurity measures

All procedures followed strict biosafety protocols in accordance with the Antarctic Treaty guidelines as well as recommendations outlined in the Scientific Committee on Antarctic Research (SCAR) Biological Risk Assessment for HPAIV in the Southern Ocean [21]. All samples collected were obtained under animal capture permits and Millennium Institute Biodiversity of Antarctic and Subantarctic Ecosystem 4/CBSCUA/2022. All procedures were approved by Instituto Antártico Chileno (INACH) (permits N° 00624/2024, 00623/2024, 00693/2024).

### Molecular detection and phylogenetic analyses

Molecular detection of influenza A virus (IAV) was performed on 24 samples from 11 skuas at the Laboratory of Molecular Virology, Pontificia Universidad Católica de Chile (LVM-UC) Although more skuas were found dead, not all of them were accessible for a safe sampling and some lacked sufficient tissue for sampling. First, sample lysis was performed with TRIzol™ reagent (Invitrogen TM 15596018) followed by RNA viral extraction with E.Z.N.A Viral RNA Kit (R6874, Omega Biotek) [11]. Then, the highly conserved matrix (M) gene of IAV was detected by RT-qPCR assay according to WHO recommendations employing the primers InfA Forward 5′ GACCRATCCTGTCACCTCTGAC 3′, InfA Reverse 5′ AGGGCATTYTGGACAAAKCGTCTA3′ Primer, and InfA Probe1 5′ FAM-TGC AGT CCT CGC TCA CTG GGC ACG-BHQ1–3′ [27]. All positive samples were subsequently tested to determine the influenza A subtype H5 clade 2.3.4.4b by RT-qPCR with the primers H5.2344-1673F TACCAAATAYTGTCAATTTATTCAAC, H5.2344-1749R GTAAYGACCCRTTRGARCACATCC, and H5.2344-1718P FAM-CTGGCAATCATDRTGGCTGGTCT-BHQ1 [27]. All One-Step RT-qPCRs were performed with AgPath-ID™ One-Step RT-PCR Reagents (4387391 Applied Biosystems™) following manufacturer’s instructions. Samples with Ct values < 37 were considered positives.

According to national guidelines and regulations, a subset of positive samples, one per individual, was submitted to the Virology and Biotechnology Laboratories of the Agricultural and Livestock Service of Chile (SAG, Lo Aguirre) for confirmation of the results. First, general detection of IAV was performed using the VetMAX™-Gold AIV Detection Kit (Applied Biosystems™, Cat No. 4485261) and subsequently, RT-qPCR assays were conducted to determine the specific lineage of H5 within clade 2.3.4.4b, following the standard operating procedures NVSL-WI-1732.02 and NVSL-WI-1767.01 from the National Veterinary Services Laboratories (NVSL) of the USDA (NVSL, 2023a; NVSL2023b).

### Genome sequencing and phylogenetic analyses

Samples with a diagnostic Ct value < 37 were selected for whole-genome amplification. The viral genome was amplified using a multi-segment One-Step RT-PCR genome amplification employing SuperScript™ III One-Step with DNA Platinum™ Taq polymerase, (12574026 Invitrogen™) with the influenza primers, Opti1-F1 5′:GTTACGCGCCAGCAAAAGCAGG-3′, Opti1-F25′:GTTACGCGCCAGCGAAAGCAGG-3′, and Opti1-R1:5′:GTTACGCGCCAGTAGAAA-CAAGG-3′ [28]. Next-generation sequencing was performed using the Oxford Nanopore Technology (ONT) platform with the Native Barcoding Kit (SQK-NBD114.96) for library preparation at LVM-UC. End-prep was performed using the NEBNext Ultra II End Repair/dA-tailing Module (E7546, NEB). Native barcodes were ligated using the Native Barcoding Expansion 96 (EXP-NBD196) and the NEB Blunt/TA Ligase Master Mix (M0367, NEB), assigning a unique barcode per sample. The barcoded libraries were pooled and purified using SPRISelect beads. Native adapters were ligated to the library using the NEBNext Quick Ligation Module (E6056, NEB), followed by purification with SPRISelect beads (B23318, Beckman Coulter) and quantification using a Qubit Fluorometer (Invitrogen™). The library was loaded onto the sequencer using the ligation sequencing kit (SQK-LSK109) according to ONT instructions for R.10.4 flow cells. Sequencing was carried out for 72 h. According to LMV-UC quality criteria, only complete sequences were submitted to GenBank.

The dataset used for phylogenetic analysis was generated from AIV sequences from avian hosts from South America and Antarctica obtained from GenBank and GISAID databases with a collection date between 1 January 2023 and 31 December 2024. MAFFT (Linux v7.526) [29] was used to align all sequences with Antarctic sequences generated in this study using only one sequence per individual. We also included some sequences from North America to provide a global context for the sequences.

The phylogenetic trees were inferred for the segments (Hemagglutinin (HA) and Neuraminidase (NA)) separately using the maximum likelihood (ML) method available in IQ-Tree3 (https://ecoevorxiv.org/repository/view/8916) with a general-time reversible (GTR) model of nucleotide substitution, incorporating a gamma-distributed rate variation among sites, selected by the BIC criterion with ModelFinder in IQ-tree software. An ultrafast bootstrap resampling process was performed with 1000 replicates to assess the reliability of each node. The result was visualized in FigTree (v1.4.4).

### Antibodies detection against influenza

First, the detection of influenza antibodies was performed for all skuas’ serum or plasma samples employing the commercial kit ID Screen® influenza A Antibody Competition Multi-species (ID.vet Cat No. FLUACA-5P) and for all positive samples, a second detection for antibodies against H5 was performed using the commercial kit ID Screen® influenza H5 Antibody Competition 3.0 Multi species (ID.vet #FLUACH5-2P). ELISA assays were performed following the manufacturer’s instructions. Positives results were identified according to the kit instructions, based on the ratio of ELISA optical densities between the specimen and the negative control (S/N). An internal positive control was included in each assay.

## Results

### Summer 2024–2025 monitoring efforts

During stationary surveillance at Harmony Point, eight adult Brown Skuas were found dead. Six carcasses lacked sufficient tissue for sampling, while the remaining two were seen before dying and exhibited neurological signs consistent with HPAIV infection, including lethargy, tremors, opisthotonus, and loss of equilibrium (Supplementary Video 1). Only one of these birds was later confirmed dead and samples were collected. This individual remained in a snowmelt stream, where its condition progressively deteriorated until its death, which occurred approximately two days after the first observation of signs. The second skua showing signs compatible with influenza was not seen again in subsequent days. Although the carcass was not retrieved, the bird was presumed dead (Supplementary Table 1).

During itinerant expeditions aboard the Betanzos and the Karpuj vessels, a total of 27 dead adult skuas were found at 50% of the visited sites, including, Cuverville (*n* = 1), Cormorant Island (*n* = 4), Biscoe Point (*n* = 2), Carvajal Station (*n* = 2), Avian Island (*n* = 6), Horseshoe Island (*n* = 6), and Lagotellerie Island (*n* = 6) (Supplementary Table 1). However, due to logistical constraints and adverse weather conditions that prevented prolonged stays at the sites, dead birds from Carvajal Station and Biscoe Point were not sampled. No skua mortality was observed at the remaining eight sites.

Most dead skua carcasses exhibited well-preserved plumage and showed no visible signs of trauma or injury. Most of them (20/35) were found in a ventral position, often with neck postures suggestive of opisthotonus at the time of death (Figure 2a,b). Two individuals had dried vomit around the beaks (Figure 2c). Carcasses without signs of predation had estimated body condition scores of 2/5 and 3/5, consistent with acute death progression. While most dead skuas appeared to have died recently, some carcasses showed evidence of scavenging or were too decomposed for sampling.

Although adults and chicks of *Pygoscelis* penguin and Antarctic Shag were observed dead, the numbers were consistent with natural mortalities regularly observed in the area, and no other evidence related with HPAIV was detected. Other live bird species, including Antarctic Terns (*Sterna vittata*) and Snowy Sheathbill *(Chionis albus*), were observed across sites. None displayed clinical signs compatible with HPAIV infection. All these birds at breeding colonies or active nests exhibited typical territorial behaviour for the breeding season.

### HPAIV A/H5 clade 2.3.4.4b is associated with skua mortality

A total of 24 samples from 11 carcasses were collected for viral detection, representing 31% of detected individuals. Samples from other carcasses [24] were either inaccessible for a safe sampling or lacked sufficient tissue. Of these, 33% were cloacal (*n* = 8), 41% orotracheal (*n* = 10), and 25% were obtained from the brain (*n* = 6).

All swabs were positive for influenza A gene M and were also positive for HPAIV A/H5 clade 2.3.4.4b lineage. All samples derived to the SAG National Reference Laboratory were confirmed according to their standardized protocols, supporting the circulation of the clade in Antarctic seabirds (Table 1). Ct values for clade 2.3.4.4b were between 12,9 in a brain sample from SP-6 (Avian Island) to 36,4 in a cloacal sample from SP-9 (Lagotellerie Island). No clear differences in Ct values were detected among sampling sites or tissue types (Table 1).

Skuas positive to HPAIV A/H5 clade 2.3.4.4b were detected in six localities, including Harmony Point (*n* = 1), Cuverville (*n* = 1), Cormorant Island (*n* = 1), Avian Island (*n* = 4), Horseshoe Island (*n* = 2), and Lagotellerie Island (*n* = 2) (Supplementary Table 1 and Figure 1). Skua population across these locations have shown to differ in admixture proportions based on genomic analyses by Jorquera et al., (2025). Specifically, individuals from most of those sampled sites exhibit admixed ancestry with a predominant genomic contribution from South Polar Skuas, whereas individuals from Harmony Point are primarily assigned to an admixed cluster with a higher Brown Skua genomic component.

**Figure 3.**
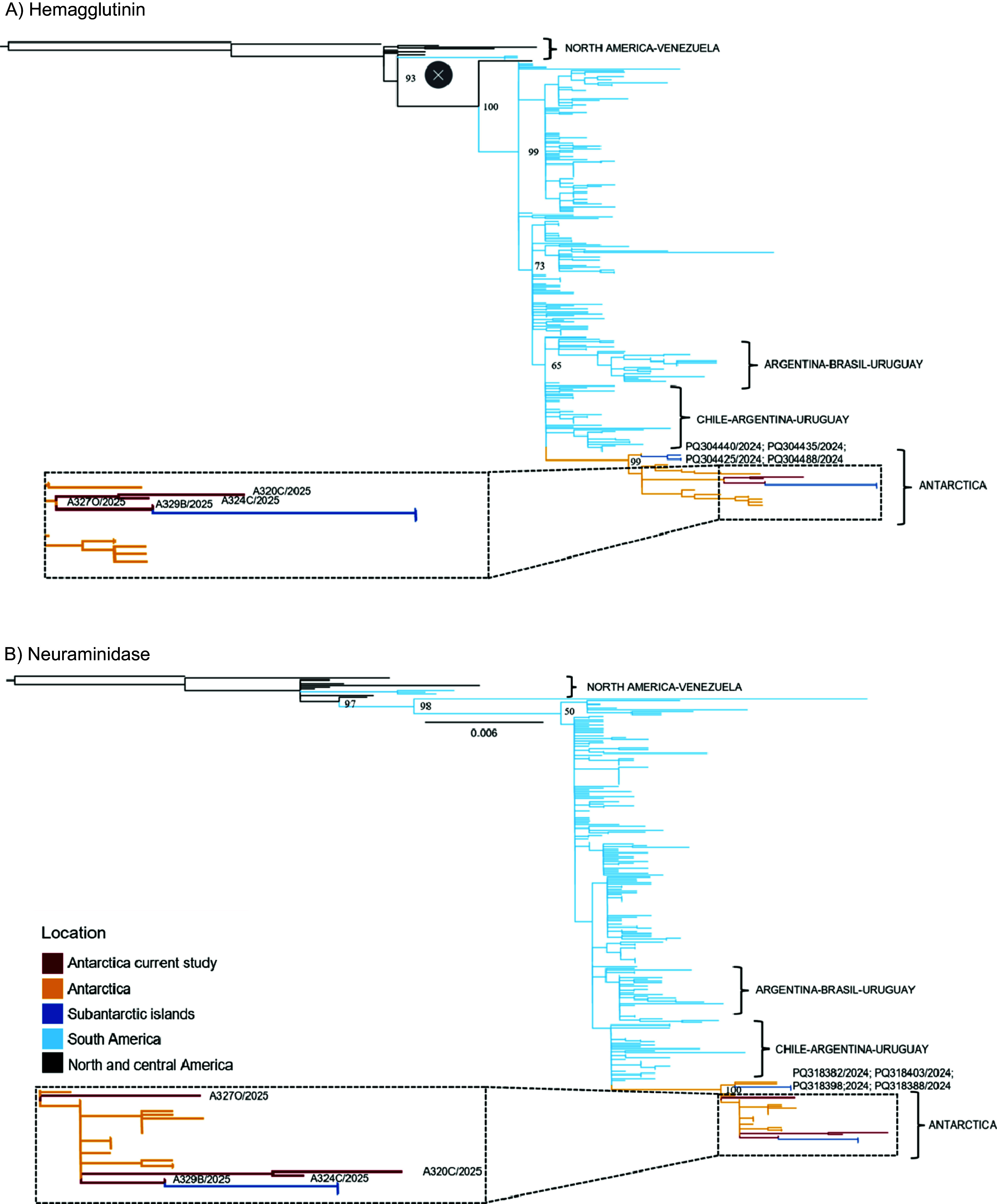
Maximum-likelihood (ML) trees were inferred for each surface protein. (a) Hemagglutinin (HA) with 296 sequences. (b) Neuraminidase (NA) with 290 sequences. The datasets include sequences from North and South America, as well as the Antarctic region, with collection dates ranging from 1 January 2023 to 31 December 2024. Ultrafast bootstrap values are provided for key nodes. Figure 3. long description.

Six complete influenza virus genomes were generated from four individuals. One sequence from Cuverville and Horseshoe Island, and two per Avian Island, were used for phylogenetic reconstruction of the HA and NA segments, ensuring one per individual (Figure 3a,b). Phylogenetic trees showed that all sequences clustered within a monophyletic clade comprising all Antarctic sequences. The sequences from this study were most closely related to viruses reported from Fildes Bay (King George Island) during the same period. Consistent with other studies, the phylogenetic structure indicates a single introduction of the influenza virus into Antarctica, likely associated with South American sources.

Serology assays to detect antibodies against influenza A/H5 were performed on serum collected from two healthy Brown Skuas captured at Harmony Point. Both individuals were seropositive for A/H5, however, none tested positive for influenza A in cloacal swabs.

## Discussion

Our surveillance identified 35 dead skuas, 11 of which tested positive for HPAIV A/H5 clade 2.3.4.4b, representing the first documented evidence of skua mortality associated with this virus beyond the Antarctic Circle (AC). These individuals were found in areas where recent reports had indicated suspected HPAIV-related mortalities. The first PCR-confirmed case in skua during the 2023–2024 season was reported on Lagoon Island by WAHIS (SCAR database, https://scar.org/library-data/avian-flu). Subsequently, several suspected skua cases were reported in the SCAR database by the IAATO during the 2024–2025 breeding season. To our knowledge, the first detailed report confirming a positive case during the 2024–2025 season was provided by this study [13], and this was later supported by additional confirmed cases in Marguerite Bay [30] and Cuverville Island [31]. These findings suggest that the current circulation of this viral clade in Antarctic seabird breeding sites may be disproportionately affecting skuas, compared with other sympatric species.

At Harmony Point, neurological signs and rapid clinical deterioration preceding death closely resemble signs described in other wild birds infected with HPAIV [25, 32]. In carcasses, the head and wing position are signs of acute death, and together with the low RT-qPCR Ct values, further support HPAIV as the most likely cause of death [21, 33, 34]. Although we did not perform differential diagnoses for other infectious agents such as avian cholera, influenza infection is known to cause fatal outcomes in closely related species such as the Great Skua [20, 25], and has also been documented in skuas from Antarctica [14]. The molecular detection of HPAIV A/H5 2.3.4.4b in skuas across Avian, Horseshoe, and Lagotellerie Islands highlights the need for geographically extensive surveillance throughout the region.

In contrast, no other sympatric seabird exhibited evident mortality or clinical signs of influenza infection, despite co-occurrence with infected skuas. No unusual behaviour or deaths were recorded for Antarctic Tern, even when it is a potential candidate for transpolar viral transmission. The same pattern was observed in Southern Giant Petrel or Snowy Sheathbill even though both species are scavengers and could plausibly become infected through consumption of contaminated carcasses [35].

The observed interspecific differences in mortality suggest that species within the genus *Stercorarius* may exhibit higher susceptibility to HPAIV infection, potentially due to the acute manifestation of the disease. No live skuas were sampled, as very few solitary individuals were observed alive and nesting. This pattern highlights the importance of prioritizing skuas for active monitoring to clarify their role in Antarctic outbreaks.

South Polar and Brown Skuas belong to the order Charadriiformes, a taxonomic group considered a major reservoir of AIVs and a key vector for long-distance viral dispersal [35]. Both species undertake seasonal migrations, but their strategies differ markedly. South Polar Skuas migrate extensively to the North Atlantic and North Pacific [32], whereas Brown Skuas generally remain within the Southern Hemisphere [33, 34]. These differences in migratory behaviour may influence their exposure to viral strains circulating at lower latitudes between the two species.

The HPAIV A/H5 infection patterns observed here parallel those recently reported for Great Skuas in the Northern Hemisphere, where severe outbreaks disproportionately affected this group. These similarities may reflect shared ecological traits, such as predation and scavenging, which increase exposure to infected birds or carcasses. Differences in trophic niche may further influence exposure risk. For example, Brown Skua predominantly preys on penguin chicks and eggs during the breeding season, whereas South Polar Skua feeds mainly on marine prey such as fish and krill [36]. These dietary distinctions likely shape their interactions with infected hosts or contaminated carcasses, potentially mediating species-specific disease outcomes [37, 38].

The HPAIV A/H5N1 clade 2.3.4.4b in the Subantarctic region was first detected in September 2023 on South Georgia Island and subsequently reported in multiple species, including kelp gulls, albatrosses, penguins, and marine mammals such as Antarctic Fur Seals (*Arctocephalus gazella*) and Southern Elephant Seals (*Mirounga leonina*) [11, 39]. Reports of this viral clade in penguins, other wild birds, and marine mammals were later documented in the Antarctic Peninsula and the South Shetland Islands [13, 14, 40]. However, genomic and serological data for skuas, as well as for other Antarctic hosts, remain insufficient to determine the transmission routes or to clarify the role of skuas in viral dispersal and propagation. In this study, we detected antibodies against A/H5 in two surviving individuals without detectable viral load, indicating prior exposure to the influenza virus. These findings highlight the need for long-term surveillance, including integrated serological and phylogeographical analyses, to better understand the pathways by which skuas become infected in the Antarctic environment.

If species within the genus *Stercorarius* are indeed highly susceptible to HPAIV infection, the demographic consequences could be detrimental. Although both species are currently categorized as ‘Least Concern’ by the International Union for Conservation of Nature (IUCN), their population trends differ. South Polar Skua remains globally stable, whereas Brown Skuas populations have shown declines that predate the HPAIV A/H5 outbreak [39]. Some efforts reveal that regional nesting dynamics across the Antarctic Peninsula exhibit heterogeneous patterns. At Ryder Bay, one of the southernmost breeding sites on the Antarctic Peninsula, long-term monitoring has evidenced a sustained increase in breeding pairs and occupied territories of South Polar Skua [36]. In contrast, the breeding population of both species has remained relatively stable at Harmony Point over the last 25 years [38]. Further north, at Signy Island (South Orkney Islands) the breeding population of Brown Skua has increased, whereas South Polar Skua has declined from 10 breeding pairs to just one [37]. Similarly, recent declines of South Polar Skua have been reported at Stinker Point, Elephant Island [41]. These fluctuations likely result from local ecological drivers such as prey availability and interspecific competition [24, 37, 42]. Unfortunately, no population estimates are available for skuas in Antarctic locations associated with reports of HPAIV A/H5. Moreover, although the devastating findings reported here, characterized by skuas mortalities and presence of solitary nesting individuals, and supported by other reports across the Antarctic Region [30, 31], suggest potential population-level impacts, the current evidence remains insufficient to confidently assess the effects of the outbreak on this species. Given that many breeding niches may already be saturated [43], additional mortality associated with HPAIV could exacerbate pressures on vulnerable populations. All these characteristics suggest that skuas should be considered a sentinel species for evaluating the introduction and evolution of influenza viruses in the Antarctic Continent.

Interspecific variation in HPAIV responses among bird species are likely the result of complex interactions among environmental factors, host characteristics, and viral traits [43]. Prior exposure to low pathogenicity AIV, for instance, may confer partial immunity and enhance host survival [44]. Moreover, although Anseriformes and Charadriiformes orders have been considered as reservoirs to AIV, substantial interspecific susceptibility variation exists. Such differences could stem not only from ecological or behavioural traits but also from underlying genomic variation influencing host immune responses [45, 46]. Comparative approaches integrating ecological and genomic data represent a valuable approach to better understanding species-specific susceptibility.

## Conclusions

The detection of HPAIV A/H5 clade 2.3.4.4b in adult skuas and the apparently higher susceptibility of Antarctic species from the genus *Stercorarius* enhances the necessity of maintaining and strengthening active surveillance across Antarctic seabird populations. Although these findings must be interpreted cautiously, enhanced monitoring, including long-term colonies censuses and population monitoring of skuas, is essential to determine the epidemiological role of skuas and to evaluate the potential demographic consequences of ongoing viral circulation.

## Supporting information

10.1017/S0950268826101733.sm001 León et al. supplementary material León et al. supplementary material

## Data Availability

The data that supports the findings of this study will be openly available in GenBank from January 2026 at https://www.ncbi.nlm.nih.gov/genbank/, accession numbers for each segment are at Supplementary Table 2.

## Supplementary material

The supplementary material for this article can be found at http://doi.org/10.1017/S0950268826101733.

## Long descriptions

### Table 1 Long description

This is a structured data table containing 8 columns and 20 rows of data organized by geographic sampling locations. The columns from left to right are: Site name with abbreviations, decimal latitude, decimal longitude, Species and Skua ID, swab sample obtained, Ct value for M gene segment, Ct value for the pathogenic clade H5 2.3.4.4b, and influenza subtype determined by nanopore sequencing (results include H5N1, NA, or an asterisk).

Navigate back to Table 1.

### Figure 1 Long description

This figure is a geographic map showing the monitoring locations along the Antarctic Peninsula included in this study. The map includes all sampled or visited sites, as well as the locations where HPAIV H5N1-positive dead skuas were found. It also identifies Antarctic Specially Protected Areas (ASPAs) and Antarctic Specially Managed Areas (ASMAs). The map includes coordinate grid around its borders, with latitude ranging from −70° to −74° and longitudes from −57° to 72°. The Antarctic Circle is represented by a dashed diagonal line, showing that some positive locations were situated south of the Antarctic Circle. A large rectangular inset in the lower right corner provides a magnified view of the southernmost area outlined by a white square on the main map. The map legend includes: Dashed Line: Antarctic Circle; Orange Dots: Sampled locations; Red Dots: HPAIV-positive locations; Pink shaded Areas: ASPAs; Yellow Shaded Areas with Outlines: ASMAs.

Navigate back to Figure 1.

### Figure 2 Long description

This figure is a composite of three photographs of dead skuas, labeled a, b and c, illustrating the positions and external appearance of the carcasses at the time they were found. Photograph a (Top-left): A dead skua lying on its side on grey rocks. The wings are partially arched or folded over its back. The carcass is in a dorsal recumbency position, with well-preserved plumage and no apparent traumatic lesions. Photograph b (Bottom left): A skua carcass in a compact or curled position. The position of the neck suggests possible opisthotonus sign at the time of dead. The plumage is also well-preserved, and there are no sign of predation or external trauma. Photograph c (Right): A carcass resting directly on a large, flat, grey boulder. A discharge or vomitus is visible on the rock immediately in the front of the head.

Navigate back to Figure 2.

### Figure 3 Long description

This figure presents maximum-likelihood phylogenetic trees of two influenza virus surface glycoproteins: A) Hemagglutinin and B) Neuroaminidase. Both trees illustrate the evolutionary relationships and geographical clustering of viral isolates. Branch colors correspond to the sampling locations defined in the legend: Dark Red: Antarctica from the current study; Orange: Antarctica (previously published sequences); Dark Blue: Subantarctic islands; Light Blue: South America; Black: North and Central America. The basal linages are composed primarily of sequences from North America and Venezuela. Withing the South American lineage, two major sub-clades are evident: one comprising isolates from Argentina-Brazil-Uruguay, and another including Chile-Argentina-Uruguay. The Antarctic clades contain all sequences generated in the present study.

Navigate back to Figure 3.
