# Supplementary material for: Seabirds’ mortalities in Antarctica caused by HPAIV: skuas as a sentinel species
Source: Epidemiol Infect. 2026 Jun 9;154:e102. doi: 10.1017/S0950268826101733 (PMC13437992; doi:10.1017/S0950268826101733)
Supplement: Supplementary file 1 [file S0950268826101733sup001.zip › Supplementary video_description.docx]

Supplementary video1: Brown Skua (individual BS1) from Harmony Point that was found at a snow melt stream with neurological clinical signs compatible with HPAIV infection. The clinical signs include mainly lethargy, tremors and loss of equilibrium. The individual was sampled after its death.
