# Supplementary material for: Seabirds’ mortalities in Antarctica caused by HPAIV: skuas as a sentinel species
Source: Epidemiol Infect. 2026 Jun 9;154:e102. doi: 10.1017/S0950268826101733 (PMC13437992; doi:10.1017/S0950268826101733)
Supplement: Supplementary file 1 [file S0950268826101733sup001.zip › Supplementary_table_1.docx]

**Supplementary table 1:** Total of dead skuas found at each site.

| **Abbreviation** | **Site name** | **Surveillance effort** | **Decimal Latitude °S** | **Decimal Longitude °W** | **Number of dead *Stercorarius* spp. birds** |
| --- | --- | --- | --- | --- | --- |
| **LR** | Lions Rump | Karpuj vessel | 62.132.683 | 58.122.040 | 0 |
| **ARI** | Ardley Island | Karpuj vessel | 62.212.797 | 58.933.383 | 0 |
| **HP** | Harmony Point | Stationary surveillance | 62.305.650 | 59.195.120 | 8 |
| **KI** | Kopaitic Island* | Betanzos vessel | 63.313.819 | 57.911.625 | 0 |
| **OB** | Bernardo O’higgins Base | Betanzos vessel | 63.320.893 | 57.899.650 | 0 |
| **THI** | Two Hummock* | Betanzos vessel | 64.151.414 | 61.753.082 | 0 |
| **CU** | Cuverville Island | Betanzos vessel | 64.793.438 | 63.967.051 | 1 |
| **LI** | Lichtfield Island | Betanzos vessel | 64.799.999 | 64.086.279 | 0 |
| **CI** | Cormorant Island | Betanzos vessel | 64.799.999 | 63.966.666 | 4 |
| **BP** | Biscoe Point | Betanzos vessel | 64.806.700 | 63.761.007 | 2 |
| **GGV** | Gabriel Gonzalez Videla Base* | Betanzos vessel | 64.823.431 | 62.857.285 | 0 |
| **PI** | Perch Island* | Betanzos vessel | 66.000.000 | 65.366.667 | 0 |
| **CS** | Carvajal Station | Betanzos vessel | 67.760.360 | 68.914.526 | 2 |
| **AI** | Avian Island | Betanzos vessel | 67.773.099 | 68.894.866 | 6 |
| **HI** | Horseshoe Island | Betanzos vessel | 67.807.993 | 67.295.349 | 6 |
| **LAI** | Lagotellerie Island | Betanzos vessel | 67.882.431 | 67.399.990 | 6 |

Notice that due to logistical and safe reasons, not all carcasses were sampled. “*” The location is not reported as a breeding site of skuas and no breeding skuas were observed.
