# Supplementary material for: Seabirds’ mortalities in Antarctica caused by HPAIV: skuas as a sentinel species
Source: Epidemiol Infect. 2026 Jun 9;154:e102. doi: 10.1017/S0950268826101733 (PMC13437992; doi:10.1017/S0950268826101733)
Supplement: Supplementary file 1 [file S0950268826101733sup001.zip › Supplementary_table_2.docx]

**Supplementary table 2:** Acquisition numbers for each segment.

| **Sp / Skua ID** | **Obtained swabs** | **Stain name** | **GenBank Database Nucleotide Access IDs** | | | | | | | |
| --- | --- | --- | --- | --- | --- | --- | --- | --- | --- | --- |
|  |  |  | **PB2** | **PB1** | **PA** | **HA** | **NP** | **NA** | **M** | **NS** |
| SP-1 | Brain | A/brown skua/Antarctic/A329B/2025(H5N1) | PX373541 | PX373544 | PX373546 | PX373547 | PX373545 | PX373542 | PX373540 | PX373543 |
| SP-3 | Orotracheal | A/brown skua/Antarctic/A324O/2025(H5N1) | PX373600 | PX373599 | PX373597 | PX373596 | PX373601 | PX373598 | PX373602 | PX373595 |
|  | Cloacal | A/brown skua/Antarctic/A324C/2025(H5N1) | PX373591 | PX373593 | PX373589 | PX373588 | PX373587 | PX373592 | PX373594 | PX373590 |
| SP-6 | Brain | A/brown skua/Antarctic/A327B/2025(H5N1) | PX373550 | PX373548 | PX373551 | PX373549 | PX373555 | PX373552 | PX373553 | PX373554 |
|  | Orotracheal | A/brown skua/Antarctic/A327O/2025(H5N1) | PX373561 | PX373557 | PX373562 | PX373559 | PX373560 | PX373563 | PX373558 | PX373556 |
| SP-7 | Cloacal | A/brown skua/Antarctic/A320C/2025(H5N1) | PX373567 | PX373571 | PX373565 | PX373566 | PX373569 | PX373568 | PX373570 | PX373564 |
